# The association of the quality of life with Afghan households’ food insecurity before and after the recent political change in Afghanistan: a comparative analysis

**DOI:** 10.1186/s12889-023-16967-z

**Published:** 2023-10-23

**Authors:** Jumakhan Saif-Nijat, Mohammad Reza Pakravan-Charvadeh, Saeed Gholamrezai, Mehdi Rahimian, Ginny Lane, Daniel Béland, Mustafa Koc, Nancy Clark, Nasrin Omidvar, Rasoul Sadeghi, Hassan Vatanparast

**Affiliations:** 1 Department of Agricultural Economics and Rural Development, Lorestan University, Khorramabad, Lorestan Iran; 2 College of Agricultural and Life Sciences, Margaret Ritchie School of Family and Consumer Sciences, University of Idaho, Moscow, ID USA; 3 Department of Political Science, McGill University, Montreal, QC Canada; 4 Department of Sociology, Centre for Studies in Food Security, Toronto Metropolitan University (formerly known as Ryerson University), Toronto, Canada; 5 Faculty of Human and Social Development, School of Nursing, University of Victoria, Victoria, BC Canada; 6 Department of Community Nutrition, National Nutrition and Food Technology Research Institute (NNFTRI), Faculty of Nutrition Sciences and Food Technology, Shahid Beheshti University of Medical Sciences, Tajrish, Velenjak, Iran; 7 Faculty of Social Science, University of Tehran, Tehran, Iran; 8 College of Pharmacy and Nutrition, University of Saskatchewan, Saskatoon, SK Canada

**Keywords:** Food security, Quality of life, Taliban’s takeover, Afghanistan

## Abstract

The overreaching objective of the current study is to investigate the association of quality of life with Afghan households’ food insecurity. The data was collected immediately after the Taliban took control of a large part of Afghanistan. About a total of 555 households’ heads participated in a face-to-face interview, using the HFIAS and WHOQOL-100 questionnaires along with some questions related to their socioeconomic characteristics at two different times, before and after the Taliban’s takeover. The comparative analysis showed that 98% of Afghan households were food insecure after the Taliban takeover, while 70% of them faced food insecurity before the Taliban’s takeover. The quality of life in the Taliban era is worse than before the Taliban. All dimensions of quality of life have decreased, and this decrease was more pronounced for the psychological, environmental, and physical domains. It is recommended that international organizations, NGOs, and local agents focus on these dimensions of the quality of life to improve food security.

## Introduction

One year after the Taliban’s return to power in Afghanistan, the deplorable situation of Afghans is worsening. The economic situation is dire, the rate of malnutrition is increasing, the rights of women are under threat, and the health care system is collapsing [1]. The Taliban leadership has created conditions that have resulted in many Afghans being compelled to provide for their basic needs through unacceptable methods, such as selling their children, selling their kidneys, and giving sleeping medicine to children to consume less food [2]. Moreover, social, cultural, and physical restrictions have caused undesirable consequences on Afghan people’s lives. All of these disruptions to established ways of life can affect overall quality of life and food security status, which requires steady economic and physical access to enough food for each person within a household to live an active, healthy life [3, 4]. Quality of life (QoL) is defined as an ‘Individual’ perception of their position in life in the context of the culture and value systems in which they live in relation to their goals, expectations, standards, and concerns [5]. The QOL consists of several important domains, including physical, psychological, environmental, social, and independence, which may affect food security status. Within the psychological domain, pain and discomfort has also been shown to affect food security through increased anxiety and stress [6–9]. Poor social relationships are also linked with food insecurity in some studies [10–16]. Many studies agree that the psychological domain is one of the most critical dimensions of the QOL associated with food insecurity [17–19]. Previous studies have confirmed the significant association of household food insecurity with quality of life among different population groups, including infants, adults, and underrepresented minorities [20]. A study confirmed that a households’ food insecurity was significantly associated with fair-to-poor general health in rural areas, but not in urban areas [21]. Food insecurity and poor mental health have been significantly associated with a low level of the QOL in Ethiopia [22]. Another study showed that children who live in food insecure households have poorer health-related quality of life (HRQOL) [22]. While previous studies have focused on the association between food insecurity and QOL, the present study explores the effect of different QOL dimensions on households’ food insecurity. Nevertheless, no studies regarding quality of life and household food insecurity in a war zone such as Afghanistan have been published. In fact, the innovation of the current study is the test of inverse association between food security and QOL. The results of the present study provide ample opportunities for international institutions and local policy makers to find the best and most accessible ways to improve the food security situation, focusing on the quality of life. Therefore, the overall goal of the current study is to investigate the impact of quality of life on Afghan households’ food insecurity before and after the Taliban’s takeover. To achieve this goal, the following sub-objectives will be investigated:

- Assess and compare the food security of Afghan households before and after the Taliban’s takeover.
- Calculate and compare the quality of life of Afghan households before and after the Taliban’s takeover.
- Assess the association of all QOL dimensions with food security status of Afghans before and after the Taliban’s takeover.

## Materials and methods

### Theoretical framework

We adopted the *Social Ecological Framework* with focusing on the accessibility pillar of food security. In this framework, we focused on the policy changes as the final layer, and also, we considered layer 1 (environmental) and layer 3 (social) to follow our main objectives. We also adopted the *Intersectionality Framework* to assist in understanding the interrelated factors of the QOL, including social, environmental, physical, phycological, spirituality, and independency that contribute to food insecurity and shape inequitable food security solutions embedded in sociopolitical and structural power relations.

**Fig. 1:**
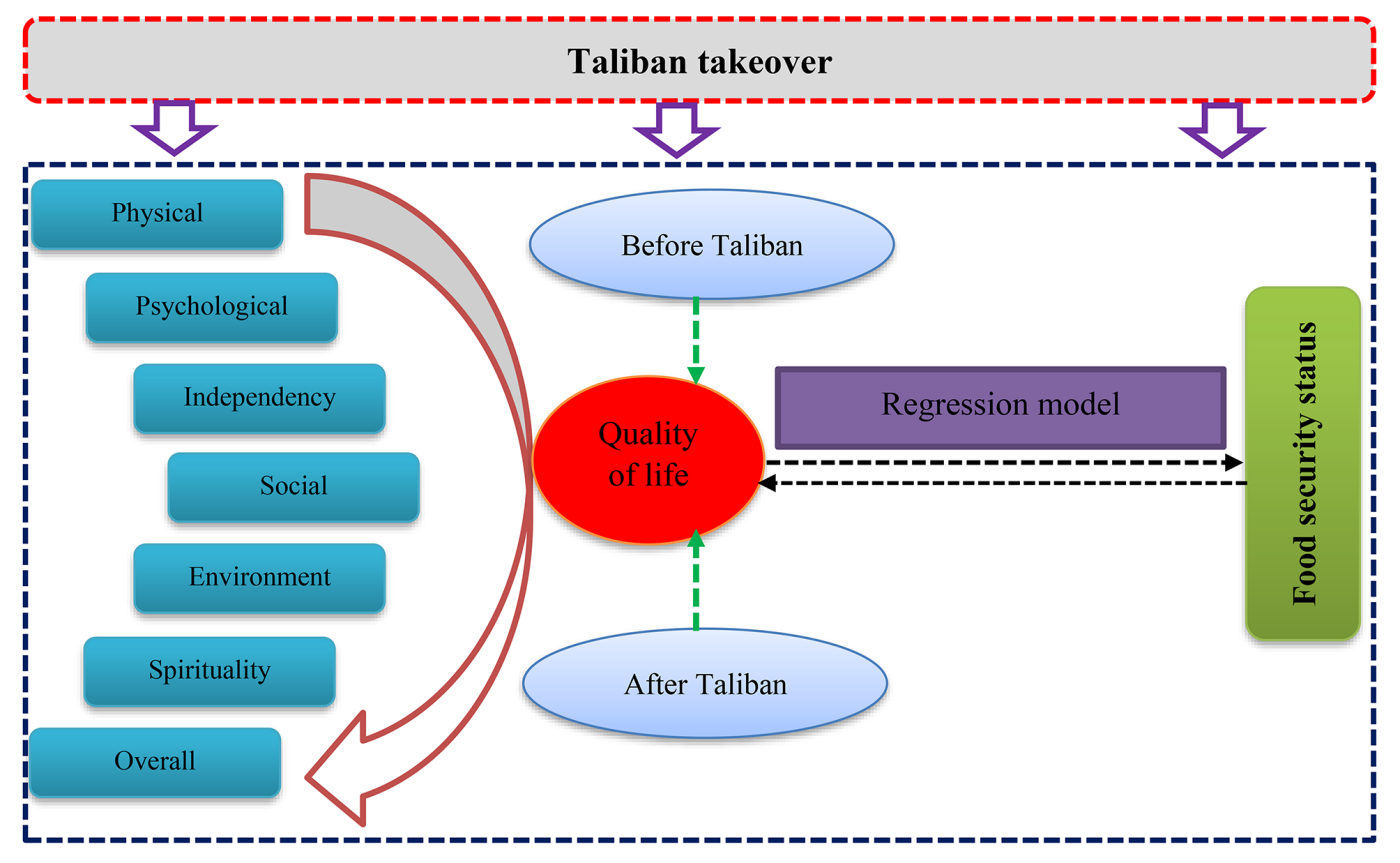
Conceptual framework of the current study

In this cross-sectional study, we investigated the association of quality of life of Afghan households with food security (Fig. 1) and vice versa. In the first stage, the data were gathered through a detailed questionnaire. Then, all domains and facets of the QOL were calculated using WHOQOL-100 method. Next, the food security situation of the participating households was calculated using HFIAS standard questionnaire. Finally, the association of all domains with food security will be determined using regression models. We also tested a bidirectional relationship between food insecurity and the QOL in Afghanistan after Taliban takeover. Figure 1 shows a conceptual framework of the current study.

### Study location

**Fig. 2:**
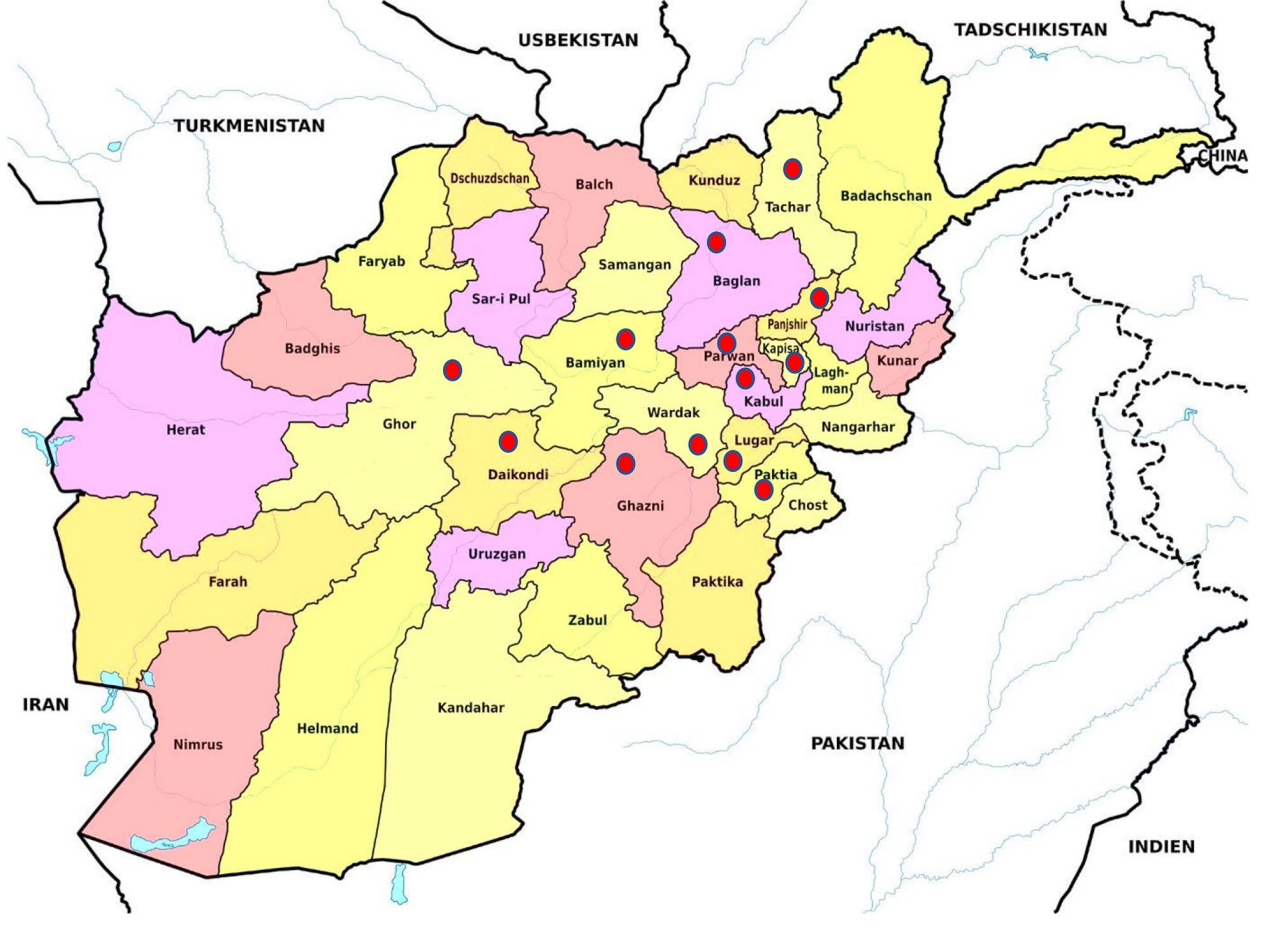
The map of Afghanistan (red circles show the locations where study conducted)

This study was carried out in Afghanistan, which is located at the crossroads of Central Asia and South Asia. Figure 2 is a map of the study location. Afghanistan is bordered by Pakistan to the East and South, Iran to the West, Turkmenistan to the Northwest, Uzbekistan to the North, and Tajikistan to the Northeast. It is the 41st largest country in the world and consists of 34 provinces. Most Afghan people belong to the Sunni religion (80%), and others are Shia (15%) and non-denominational (5%).

The capital city of Afghanistan is Kabul, where about 4.44 million people live. The recent Taliban takeover led to their return to power after 21 years. To have a representative sample of Afghan people, we collected data from all geographical locations in Afghanistan; however, due to intense military conflict and strict control of the western regions, it was not possible to complete the questionnaire in those regions. Therefore, data were gathered from Afghan families living in the Central (Kabul, Bamyan, Parwan, Panjshir, Daykundi, Kapisa, and Wardak), North (Baghlan), South (Paktia, Ghazni, Logar), North East (Tachar), and North West (Ghor) provinces.

### Data collection process and study population

A multi-stage stratified sampling method was used whereby provinces were selected based on their low level of military conflict, then locations were selected based on the interviews’ availability to collect the data in the area. The sample size was determined by G-power software version 3.1.9.4 with level of significance 0.05 and statistical power 0.95. Using the probability sampling method, about 555 questionnaires were filled out by Afghan families. Due to the critical situation in Afghanistan during Taliban takeover, our interviewers determined the random sampling using house plaque of Afghan households (stratum) in each area. To support consistent data collection, heads of Afghan households were interviewed. Interviewees were then randomly selected from families living in the selected areas. The questionnaire had three sections, including the HFIAS to measure food insecurity, the WHOQOL-100 to calculate quality of life, and a section on the socioeconomic determinants. The questionnaire was administered through face-to-face interviews, during home visits to selected households. Before collecting the data, 10 Afghan students with different educational backgrounds, including agricultural economics, health economics, public health, and nutrition sciences, were recruited, and trained to administer and enter responses into the questionnaires. They learned how to communicate with local people, fill out the questionnaire, and perform the data cleaning process. We also assigned an Afghan student as the principal leader of the student interviewers. The Taliban invaded Afghanistan on 15 August 2021, and it took about three months to occupy the country. We started the data collection process five months after the Taliban’s takeover from January to April 2022. We asked the respondents to answer the questions raised in the designed questionnaire in the conditions before and after the deployment of Taliban forces. Also, the designed questionnaire was translated into Dari and Pashto language and we recruited Afghan students from both of these languages.

### Food insecurity measurement

Household Food Insecurity Access Scale (HFIAS) was used to measure Afghan households’ food insecurity. This standard tool is used to calculate household access to food at the household and national level. The HFIAS questionnaire is used to assess the prevalence of household food insecurity (emphasis on the access dimension) and to monitor change in food insecurity during a certain period [23]. The HFIAS uses a set of nine questions, which has been proven to be effective in separating food secure from food insecure households across the world. This instrument has been used previously with Afghan people [24–27] and the Persian version has been validated [28–30]. The HFIAS measures three different dimensions of household food insecurity; the first question asks about uncertainty and concern of the family in providing the food needed; the second to fourth questions focus on insufficient quality of household food, including variety and food preferences; and the fifth to ninth questions ask about the intake of insufficient food and its physical consequences [23]. It is to point out that questions related to the quality of household food are not directly related to nutritional quality. According to the answers given to the nine questions of the HFIAS questionnaire, participating households can be categorized into four different levels, including food secure, and marginally, moderately, and severely food insecure [25, 26]. The household HFIAS food insecurity score is calculated by summing the scores for all of the nine questions. Thus, the maximum score can be 27 (answering “always” to all questions), and the minimum score will be zero (answering “no” to all questions or not answering the questions). Based on this coding, a higher score indicates greater food insecurity, and a lower score is equivalent to less food insecurity [31, 32].

### Measurement of the quality of life

To calculate quality of life, the WHOQOL-100 measurement tool was used. It encompasses in a sophisticated manner physical health, psychological status, level of independence, social relationships, personal beliefs, and their relationships with features of the environment. This tool consists of 100 questions that ask how a person feels about his/her quality of life, health, and other areas of life. After asking all 100 questions, all questions are sorted into six domains, including physical, psychological, independence, social, environment, and spiritual. This is based on four questions per facet, for 24 facets of quality of life. In addition, four questions^1^ address Overall quality of life and general health. Domain and facet scores are scaled in a positive direction where higher scores denote higher quality of life. Some facets (pain and Discomfort, Negative Feelings, dependence on medication) are not scaled in a positive direction, meaning that for these facets higher scores do not denote higher quality of life. Thus, a total quality of life score derived by summing data from all WHOQOL items is not recommended.

Domain scores are the means of all question scores in that domain multiplied by four and range between 4 and 20. After calculating the domain scores, they are converted into a unique index between 0 and 100 to facilitate comparison of all indexes. This questionnaire was validated in several countries, and the reliability and validity of the Persian version were confirmed in Iran [33]. Due to the similarity between languages spoken in Iran and Afghanistan, we used the Persian version. To ensure that this tool is appropriate for the Afghanistan context, the instrument’s reliability was assessed using Cronbach’s *α* for internal consistency. For structural validity, we checked the correlation coefficients between all items constituting each subsection under each domain using Pearson’s correlation coefficients, the correlation between the subsections and their domains, and the correlation between the domains and the overall indicator of the QOL.

### Statistical analysis approach

#### Data cleaning process

To address the study objectives, we used several statistical techniques. First, data cleaning process was carried out to find typos and invalid or missing data, inconsistent data, duplicate data, and irrelevant data. For achieving the best cleaning data, we followed four steps immediately after data collection process, including inspection and profiling, cleaning, verification, reporting. After checking accuracy, completeness, consistency, integrity, timeliness, uniformity, and validity of collected data, statistical analysis was started.

### Descriptive analysis

First, the descriptive statistics were reported as minimum, maximum, means, and standard deviation for continuous factors, and frequency, percentage, and mode for categorical variables. Second, the ${\chi }^{2}$ test was used to investigate the difference between four food security categories (food secure, marginally, moderately, and severely food insecure) before and after the Taliban’s takeover. Third, due to using a continuous indicators of food insecurity and the QOL in the next step, we applied a t-test to show the difference between this indicator before and after the Taliban’s takeover. Third, Cronbach’s α and Pearson coefficients were used to check the reliability and validity of the QOL questionnaire. The data analysis of the current study was carried out using Statistical Package for the Social Sciences, Version 25. For estimation of the regression models, EViews 13 was used.

### Statistical model

Finally, regression analysis with a dummy variable was used to show the association of the QOL domains with Afghan households’ food insecurity and to rank these domains based on the coefficients and vice versa. We used two regressions to follow the objectives: first regression equation is below:

1 $$Lndomain={C}_{1}+{C}_{2}lnFI$$

Ln (domain) shows the logarithm of each domain of the QOL, C_1_ is the intercept of the regression, C2 is slope, and ln (FI) is the logarithm of food insecurity indicator. We used the logarithm of each variable to calculate the elasticity of independent variables. To calculate the change of QOL domains due to the change of food insecurity, we added a dummy variable (1 = After the Taliban’s takeover and 0 = Before Taliban’s takeover) into the intercept and the slope of the regression as follows:

2 $$Lndomain={C}_{1}+{C}_{2}LnFI+{C}_{3}TT+{C}_{4}TT*LnFI$$

TT is the dummy variable showing before and after the Taliban’s takeover. To extract the marginal effect of the food insecurity of Afghan households on each domain and overall score, the elasticity can be used:

3 $$\frac{Lndomain}{LnFI}={C}_{2}+{C}_{4}*TT$$

This equation shows the percent change of each domain due to 1% change in food insecurity status. The second regression was used to test the bidirectional association of food insecurity and the domains of the QOL as follows:

4 $$LnFI={\beta }_{1}+{\beta }_{2}LnDomain$$

*B*_*1*_ is the intercept of the regression, *B*_*2*_ is the slope, and the domain shows the different components of the QOL. To calculate the change of FI due to the change of all domains, the following equation was used:

5 $$LnFI={\beta }_{1}+{\beta }_{2}LnDomain+{\beta }_{3}TT+{\beta }_{4}TT*LnDomain$$

Baes on the Eq. 5, the elasticity can be used:

6 $$\frac{LnFI}{LnDomain}={\beta }_{2}+{\beta }_{4}*TT$$

This equation shows the percent change of food insecurity due to 1% change in each domain of the QOL.

## Results

### Descriptive analysis

Table 1 shows the descriptive analysis of the interviewed households’ characteristics. The average age of the households was 47 years and the interviewed households had an average of three children in their family. Also, the respondents had at least one employed member and one educated member, and these households had an average of two male and one female children in their families. The average area of the houses of the interviewed households was 220m^2^ and the residence period of these households was 30 years.

**Table 1:** Descriptive analysis of Afghan households’ characteristics

Variable	Descriptive			
	Min	Max	Mean	SD
*I. Continuous*				
**I-1- Household’s members characteristics**				
(a). Age of head of household (year)	22	83	47.7	12.63
(b). Household size (based on the number of children)	1	9	3.73	1.461
(c). Number of employed members	0	7	1.83	0.86
(d). Number of educated members	0	6	0.99	1.03
(f). Number of male children	0	8	2.03	1.37
(g). Number of female children	0	6	1.58	1.29
**I-2- Household’s property characteristics**				
(h). Monthly income (U.S. $)	0	1719	120	108
(i). House area (m^2^)	3	3000	220	290
(j). Distance to city center (Kilometer)	3	240	10.7	26.7
(k). Length of stay (year)	1	83	30	31.4
	Descriptive			
*II. Categorical*	Category	Frequency	Percentage	Mode
**(a). Household head’s occupation status**	1–3			
1. Employed		284	51.2	□
2. Unemployed		144	25.9	
3. Seasonal		127	22.9	
**(c). Head’s non-communicable diseases**	1–2			
1. Yes		74	13.4	
2. No		481	86.6	□
**(d). Smoking status of the head of household**	1–2			
1. Yes		117	21.1	
2. No		438	78.9	□
**(e). Household head’s education status**	1–2			
1. Illiterate		212	38.2	
2. Literate		343	61.8	□
**(f). Mother’s education status**	1–2			
1. Illiterate		380	68.4	□
2. Literate		175	31.6	
**(g). Head’s religion**	1–2			
1. Suni		385	69.3	□
2. Shia		170	30.6	
**(f). Personal saving**	1–2			
1. Yes		130	23.4	
2. No		425	76.6	□
**(g). Head’s gender**	1–2			
1. Male		527	94.9	□
2. Female		28	5.1	
**(h). Ethnicity**	1–9			
1. Uzbeks		44	7.9	
2. Baloch		3	0.5	
3. Pashtun		107	19.3	
4. Pashayee		5	0.9	
5. Tajik		194	35	□
6. Turkmens		6	1.1	
7. Sadat		7	1.3	
8. Arab		8	1.4	
9. Hazaras		181	32.6	

Participating households were on average 10 km away from the city center. According to the descriptive analysis, approximately 51% of heads of the interviewed households were employed, while 26% of them were unemployed. About 13% of heads of households were suffering from non-communicable diseases and 21% of interviewed households were smokers. Also, 38% of heads of the participating households and 68% of mothers were illiterate. Approximately 69% of the heads of households are Sunni and 76% of the responders did not have any personal savings. Finally, about 95% of heads of the interviewed households were male and most of the households were Tajik (35%).

### Validity and reliability of the QOL questionnaire

Cronbach’s $\alpha$ coefficient, ranging from 0.7 to 0.9, was applied to assess the reliability of the research tools. As Table 2 shows, this coefficient was used in two different steps. First, the reliability of each subsection was assessed using their items. The result showed that Cronbach’s $\alpha$ coefficient of all subsections was more than 0.7 which confirms the reliability of using these items within different domains. Second, Cronbach’s $\alpha$ coefficients of all domains were more than 0.7 which confirms the reliability of using these domains to calculate the overall score of the QOL. To check the validity of the questionnaire, Pearson coefficients were applied in two steps. First, the Pearson correlation coefficients between each subsection and its’ domain were calculated.

**Table 2:** The reliability test of different facets and domains of the quality of life in Afghanistan

Domains and subsections	Cronbach’s α	Pearson correlation coefficient with sub-group score	Pearson correlation coefficient with overall score
**Physical Capacity**	**0.80**		0.535^***^
Pain and discomfort	0.75	-0.741^***^	
Energy and fatigue	0.76	0.882^***^	
Sleep and rest	0.78	0.774^***^	
**Psychological**	**0.79**		0.717^***^
Positive feeling	0.82	0.592^***^	
Thinking, learning, memory and concentration	0.74	0.837^***^	
Self-esteem	0.84	0.662^***^	
Bodily image and appearance	0.75	0.708^***^	
Negative feeling	0.77	-0.560^***^	
**Level of Independence**	**0.80**		0.610^***^
Mobility	0.72	0.725^***^	
Activities of daily living	0.79	0.794^***^	
Dependence on medicinal substances and medical aids	0.75	-0.649^***^	
Work capacity	0.78	0.831^***^	
**Social Relationships**	**0.85**		0.714^***^
Personal relationships	0.81	0.816^***^	
Social support	0.79	0.792^***^	
Sexual activity	0.72	0.613^***^	
**Environment**	**0.79**		0.580^***^
Freedom, physical safety and security	0.73	0.438^***^	
Home environment	0.78	0.459^***^	
Financial resources	0.81	0.754^***^	
Health and social care: accessibility and quality	0.74	0.654^***^	
Opportunities for acquiring new information and skills	0.73	0.787^***^	
Participation in and opportunities for recreation/ leisure	0.81	0.741^***^	
Physical environment (pollution/noise/traffic/climate)	0.75	0.648^***^	
Transport	0.78	0.735^***^	
**Spirituality/Religion/ Personal Beliefs**	**0.72**		0.460^***^
Spirituality/religion/personal beliefs	0.79	1.00^***^	

Note: ^*** ,^ p < .001

The result showed that all coefficients were significant confirming these subsections have the necessary validity to measure the domains. Second, the Pearson coefficient between each domain and overall score supports the validity of the questionnaire to calculate the QOL indicator.

### Quality of Life assessment

**Fig. 3:**
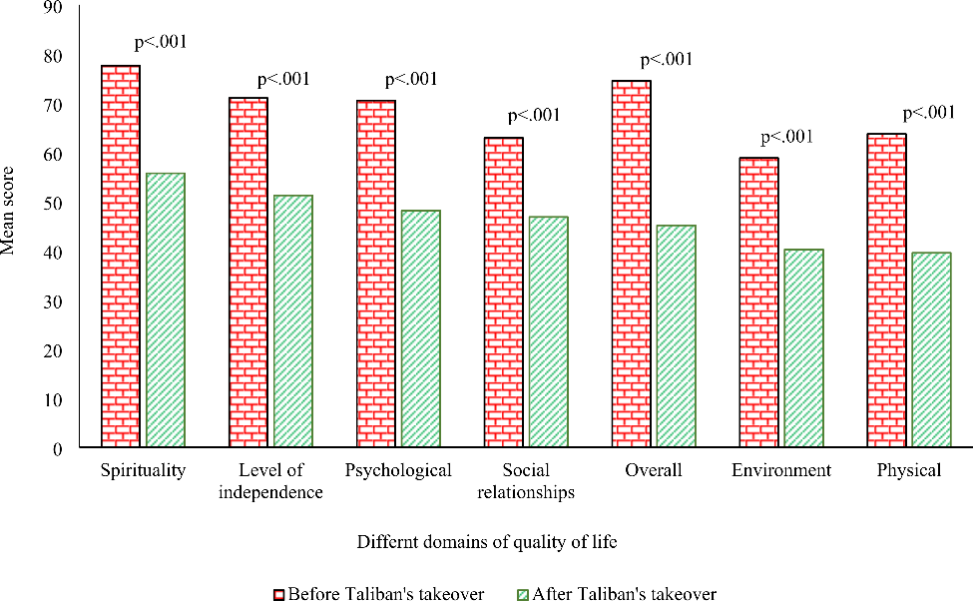
The difference of mean score of the QOL domains before and after Taliban’s takeover

After verification of validity and reliability of the questionnaire, QOL domain scores and the overall score were calculated for the two time points, as per Table 3. Based on the results, quality of life has worsened in all dimensions after the Taliban’s takeover. The physical, environmental, and psychological dimensions of the QOL had the most significant difference after the Taliban’s takeover compared to before the takeover. Also, all indicators were significantly different at the two time points. The overall QOL score differed considerably between before and after the Taliban’s takeover, with quality-of-life worsening (39% decrease) after the political change. Figure 3 shows the difference between the QOL domains at the two time points. The overall QOL score changed the most compared to individual domains.

**Table 3:** Change in the domain subsections of the quality of life of Afghan households before and after and Taliban’s takeover

Facets and Domains	Mean (Before Taliban)	Mean (After Taliban)	Difference	Change (%)	t-value
**Physical capacity**	**63.7**	**39.5**	**-24.20**	**-38**	**-32.33** ^***^
Pain and discomfort	46.44	66.48	20.04	43	19.6^***^
Energy and fatigue	64.04	36.51	-27.53	-43	-30.4^***^
Sleep and rest	73.38	48.51	-24.87	-33	-26.1^***^
**Psychological**	**70.5**	**48.1**	**-22.40**	**-31**	**-31.58** ^***^
Positive feeling	64.57	33.79	-30.78	-47	-26.6^***^
Thinking, learning, memory and concentration	68.87	46.43	-22.44	-32	-23.8^***^
Self-esteem	78.95	63.95	-15.72	-20	-19.4^***^
Bodily image and appearance	79.42	62.67	-16.75	-21	-20.3^***^
Negative feeling	39.44	65.49	26.05	66	22.6^***^
**Level of independence**	**71**	**51.2**	**-19.80**	**-28**	**-27.83** ^***^
Mobility	66.02	51.14	-14.88	-22	-17.9^***^
Activities of daily living	66.16	40.06	-20.10	-30	-26.9^***^
Dependence on medicinal substances and medical aids	30.11	47.74	17.63	58	18.3^***^
Work capacity	81.91	55.30	-26.61	-32	-23.4^***^
**Social relationships**	**62.9**	**46.8**	**-16.10**	**-25**	**-20.30** ^***^
Personal relationships	71.34	51.83	-19.51	-27	-20.5^***^
Social support	59.35	41.21	-18.14	-30	-17.6^***^
Sexual activity	57.98	47.24	-10.74	-18	-13.5^***^
**Environment**	**58.8**	**40.2**	**-18.60**	**-31**	**-26.85** ^***^
Freedom, physical safety and security	47.87	43.81	-4.06	-8	-3.8^***^
Home environment	66.85	52.98	-13.87	-20	-15.3^***^
Financial resources	55.29	27.99	-27.30	-49	-24.4^***^
Health and social care: accessibility and quality	60.59	42.69	-17.90	-29	-19.1^***^
Opportunities for acquiring new information and skills	62.56	34.32	-28.24	-45	-26.3^***^
Participation in and opportunities for recreation/ leisure	61.44	40.87	-20.57	-33	-18.6^***^
Physical environment (pollution/noise/traffic/climate)	61.45	46.26	-15.19	-24	-19.6^***^
Transport	54.16	32.65	-21.51	-23	-20.2^***^
**Spirituality/religion/ personal beliefs**	**77.6**	**55.7**	**-21.90**	**-28**	**-21.16** ^***^
Spirituality/religion/personal beliefs	77.59	55.75	-21.84	-28	-21.1^***^
**Overall quality of life and general health perceptions**	**74.5**	**45.1**	**-29.40**	**-39**	**-27.02** ^***^

Note: ^*** ,^ p < .001

Table 3 also shows the average QOL domain subsection scores of Afghan households and the change between the two time points. Within the physical capacity domain, about 40% of Afghan people’s energy decreased after the Taliban’s takeover, while pain and discomfort increased by 40%. In the psychological dimension, Afghans’ negative feelings of contentment, balance, peace, happiness, hopefulness, joy and enjoyment of the good things in life increased by 66% after the Taliban’s takeover. Also, work capacity, a subsection of the independence domain, decreased by 32%, while dependence on medicinal substances and medical aids increased by 58% after the Taliban’s takeover. The most significant decreases in the social relationship domain were related to social support and personal relationships, with 30% and 27% decreases, respectively.

Finally, a decline in financial support and opportunities for acquiring new information and skills play a crucial role in reducing the environmental domain score. According to the analysis, there was a significant difference between all subsections and domains at the two timepoints. In addition, as per Table 4, Afghan household food insecurity rose from 70% prior to the Taliban takeover to 98% after the Taliban’s takeover. Also, the percentage of households with severe food insecurity was more than doubles after the Taliban takeover.

**Table 4:** Afghan households’ food insecurity after and before Taliban’s takeover

Food security status	Number		Percent		χ^2^
	Before	During	Before	During	
**Food secure**	171	10	30.81	1.80	16.73^***^
**Marginal food insecure**	48	6	8.65	1.08	42.82^***^
**Moderate food insecure**	109	87	19.64	15.68	62.29^***^
**Severe food insecure**	227	452	40.90	81.44	79.41^***^
	**Before Taliban’s takeover**		**After Taliban’s takeover**		**t-value**
**Mean score of the Food security index**	4.72		16.0		37.87^***^

Note: ^*** ,^ p < .001

According to the output of the estimated regression in Table 5, food insecurity indicators was indirectly and significantly associated with all domains of the QOL. The food insecurity indicators had a stronger correlation with overall score and physical domain, while food insecurity had a weaker correlation with social and environmental domains.

**Table 5:** The association of food insecurity with the QOL domains after the Taliban takeover

Variable	Ln (Physical)	Ln (Psychological)	Ln (Independence)	Ln (Social)	Ln (Environment)	Ln (Spirituality)	Ln (Overall)
Intercept	4.11***	4.22***	4.23***	4.09***	4.02***	4.30***	4.25***
TT	-0.32***	-0.22***	-0.18***	-0.20***	-0.21***	-0.22***	-0.57***
Ln (Food insecurity)	-0.009***	-0.01***	-0.01***	-0.01***	-0.01***	-0.01***	-0.01***
TT*Ln (Food insecurity)	-0.07***	-0.05***	-0.05***	-0.03***	-0.04***	-0.05***	-0.17***
F-statistics	223.60***	306.8***	219.6***	127.8***	242.8***	100.7***	37.3***
Log Likelihood	-484.6	-8.1	-87.9	-248.3	-124.4	-557.5	-222.1
Durbin-Watson	1.90	1.60	1.57	1.83	1.81	1.65	1.94
Effect	-0.079	-0.066	-0.062	-0.041	-0.051	-0.061	-0.171

Note: ***, p < .001

The invers association of food insecurity with the QOL has been confirmed in the previous studies. Therefore, we assessed the bidirectionality between food insecurity and the QOL domains. The modulating association of the QOL domains with food insecurity are reported in Table 6. Psychological and environmental domains had the most significant correlation with food insecurity. A 1% improvement in the psychological and environmental dimensions of the QOL would lead to a decrease of about 1.18% and 1.09%, respectively, in food insecurity level. On the other hand, a 1% increase in the spirituality domain would only lead to a 0.14% reduction in food insecurity, lowest effect compared to other domains. Overall, a 1% increment to the overall QOL score would support a 0.10% reduction in food insecurity.

**Table 6:** The association of the QOL domains with food insecurity after the Taliban takeover

Variable	Model (1)	Model (2)	Model (3)	Model (4)	Model (5)	Model (6)	Model (7)	Effect
Intercept	9.49^***^	20.03^***^	-13.67^***^	14.34^***^	16.37^***^	9.71^***^	11.41^***^	
TT	-3.59^***^	-16.99^***^	15.35	-9.44^***^	-9.86^***^	-6.66^***^	-8.57^***^	
Ln (Physical)	-2.07^***^							**-1.03** ^***^
Ln (Psychological)		-5.68^***^						**-1.18** ^***^
Ln (Independency)			-13.58					**-0.66** ^***^
Ln (Social)				-3.50^***^				**-0.63** ^***^
Ln (Environment)					-4.06^***^			**-1.09** ^***^
Ln (Spirituality)						-2.26^***^		**-0.14** ^***^
Ln (Overall)							-2.69^***^	**-0.10** ^***^
TT*ln (Physical)	1.40^***^							
TT*ln (Psychological)		4.50^***^						
TT*ln (Independency)			12.92^***^					
TT*ln (Social)				2.87^***^				
TT*ln (Environment)					2.97^***^			
TT*ln (Spirituality)						2.12^***^		
TT*ln (Overall)							2.59^***^	
**F-statistics**	161.13^***^	223.54^***^	206.33^***^	186.67^***^	212.27^***^	161.92^***^	179.31^***^	
**Log Likelihood**	-2381.49	-2319.60	-2333.98	-2355.36	-2330.36	-2380.67	-2362.77	
**Durbin-Watson**	1.68	1.67	1.69	1.69	1.71	1.68	1.69	

Note: ^***^, p < .001

## Discussion

Afghanistan has faced many political challenges in the last decade, and the recent arrival of the Taliban government is one of the most jarring political and governmental changes. This event has dramatically affected the lives of Afghan people, including their food security. In the present study, we sought to determine the relationship between the QOL domains, subsections and food insecurity of the Afghan people. First, we found that the food insecurity of Afghan households was worse after the Taliban takeover than before the takeover. Second, all domains and facets of the QOL during the Taliban takeover are lower than before the Taliban. Finally, we found a bidirectionality between food insecurity and the QOL in Afghanistan. Considering the special conditions and current laws governing Afghanistan, the present study is likely the first research project on this subject in Afghanistan, and can be considered an innovative study to show the bidirectionality relationship between food insecurity and the QOL.

Based on the results, after the arrival of the Taliban, there has been a significant increase in the percentage of Afghan households faced various levels of food insecurity and severe food insecurity also doubled compared to the pre-Taliban period. While the UN World Food Programme reported that 93% of Afghan households were food insecure in 2022 (a 13% increase compared to before the Taliban’s governance) [34], the present study shows that the Taliban’s takeover has led to a 40% increase in food insecurity among Afghan families. Some studies reported the negative effect of conflict on food security status previously [35].

Our results showed that Afghan families’ QOL decreased after the Taliban’s takeover. The greatest decreases occurred in the physical, environmental, and psychological dimensions, respectively. The QOL physical domain decreased by 38% after the Taliban’s takeover. Within the physical domain, pain and discomfort increased by 43%, and energy and sleep decreased by 43% and 33%, respectively, after the Taliban’s takeover. Psychological and environmental domains decreased approximately 31% after Taliban takeover. In psychological domain, negative feeling increases about 66% after Taliban. This result shows that most of people don’t have a good feeling with the political change, and also approximately 32% reduction has happened in thinking, learning, memory and concentration. In environmental domain, 49% and 45% reduction have happened in financial resources, and opportunities for acquiring new information and skills. Due to sanctions imposed by international societies on Taliban, the local government face arduous economic conditions, and these restrictions along with the lack of financial resources might put pressure on the income and economic situation of the people and have led to the reduction of the financial resources of the residents of Afghanistan. On the other hand, the closure of some businesses by the Taliban and their unwillingness to continue women’s education might reduce people’s desire to increase knowledge and awareness and obtain updated information. In level of independency, dependence on medicinal substances and medical aids has had the greatest increase after Taliban takeover. Due to the difficult living conditions and the concern of many households about the uncertain future of Afghanistan, and the increase in pressure and stress on women and children, the use of medical drugs might increase.

Finally, previous studies demonstrated that food insecurity is significantly and indirectly associated with the QOL [22, 36–38] which was confirmed in the current study. None of these studies assess the association of food insecurity with the QOL domains. In fact, we showed that there is a bidirectional relationship between food insecurity and the QOL. According to our results, the low level of the QOL domains have led to increase in food insecurity level of Afghan households during Taliban regime. The strongest probable association of the QOL domains with food insecurity was related to the psychological domain. Negative feelings increased by 66% after the Taliban’s takeover. A negative feeling toward despondency, guilt, sadness, tearfulness, despair, nervousness, anxiety and a lack of pleasure in life can lead to a reduction in self-confidence [39], lack of concentration, a decrease in the possibility of achieving relaxation, and an increase in stress, which ultimately increases food insecurity. A negative feeling towards life events leads to a decrease in life expectancy and efforts to improve the country. The negative feelings registering an increase and positive feelings a decrease as food insecurity increased from mild to severe. The second main QOL domain that may affect the food insecurity of Afghan households was the environmental domain. Improving environmental capital will play a crucial role in ameliorating food insecurity in Afghanistan. Within the environmental domain, financial resources, recreation, and leisure, and acquiring new information and skill subsections had the most significant decreases after the Taliban’s takeover. Many studies reported that economic conditions are an essential determinant of food security in different locations [4, 25, 26, 40–47]. Economic and financial factors are the main aspects of the accessibility dimension of food security, especially among poor households [24, 48]. Improvement of economic and financial conditions lead to an increase in the family’s ability to improve their livelihood, as well as an increase in the ability to purchase various types of food and improve their diet quality [15, 27, 32]. Participation in and opportunities for recreation/ leisure was another important subsection of the QOL environmental domain which can affect the food insecurity of Afghan households. Having enough time for recreation and leisure leads to improved quality of life, as well as reduced anxiety and stress, and ultimately to reduced food insecurity. A study claimed that leisure may provide a way for women to embody and resist the inequitable gendered roles among women bean producers and can support enhanced food security and health [49]. The next important subsection of the QOL environmental domain that can affect food insecurity was the acquiring new information and skills by Afghan people. Acquiring new, up-to-date, and practical skills is considered an important factor to improve people’s income levels, which ultimately leads to improved food security. Also, these new skills and updated information can be acquired in different contexts, including agricultural activities and cooking. Agricultural teaching approaches employed in secondary schools have been shown to positively contribute to skills development leading to increased food security [50]. In contrast, another study showed that food skills had a limited association with food security and dietary diversity [51]. According to our results, the next strongest correlation was related to physical domain. Food insecurity might decrease the level of energy and fatigue [52], and increase pain and discomfort which finally decrease the physical domain of the QOL. On the contrary, food insecurity might lead to increase in the time of working to earn more money and finally decrease sleep and rest time. In the current study, we found a bidirectional association between the QOL and food insecurity. In fact, low level of the QOL leads to decrease in the level of food security. Implementing policies to increase energy and life expectancy may enhance the physical and social capital of Afghan families and decrease their food insecurity. Studies have found that depressive symptoms and chronic pain significantly predicted food insecurity [53, 54], while another study reported that policies reducing food insecurity may lower the incidence of chronic pain [55]. Such observations suggest a reciprocal relationship between chronic pain and food security. Pain may reduce quality of life, and trigger or exacerbate substance abuse, anxiety and depression [55], which have been identified as critical contributors to food insecurity [7]. Inadequate sleep is another important problem of the QOL physical domain of Afghan people after the Taliban’s takeover. Poor sleep quantity and quality may predispose FI adults to adverse health outcomes [56]. Addressing food insecurity may be an effective public health intervention for improving sleep quality and overall well-being in older age [57]. A study contended that sleep disorders have strong associations with depression and anxiety, also leading to significant decrease in food security [58].

Although the results showed that three domains, psychological, environmental, and physical domains, had the strongest correlation with food insecurity among Afghan people after the Taliban’s takeover, other domains, including level of independence, social relationships, and spirituality, were significantly and indirectly associated with food insecurity after the installation of the Taliban government. In fact, improvement of these factors may lead to enhancing food security of Afghan families.

## Conclusions and policy implications

Political transitions in various countries have always had beneficial and unfavorable consequences on people’s lives. The latest international political change is related to the Taliban domination of Afghanistan.

The results showed that the rate of food insecurity increased after Taliban takeover. Therefore, it is recommended that the local forces, international organizations, and NGOs increase their efforts to improve the nutrition and food security of Afghan people. Allocating food packages; expanding the availability of food in different regions of the country, especially in rural and deprived areas; efforts of developed countries to conduct negotiations with the Taliban forces to implement policies to eliminate malnutrition and food insecurity may assist with addressing food insecurity in Afghanistan.

The results also demonstrate that the quality of life in the post-Taliban period was worse than before the Taliban. Due to the severe reduction of all components of the quality of life, the government should consider implementing constructive programs to increase citizens’ vitality and positive feeling towards the future of the country, to increase the overall wellbeing and happiness of Afghan households, and to increase energy and look positively towards the future.

NGOs and international organizations should consider the three important and effective QOL domains - psychological, environmental, and physical, as investment priorities in order to increase quality of life, and finally decrease in food insecurity level. Implementation of policies to increase and improve Afghan people’s quality of life, investment in recreation and tourism, increased humanitarian financial and economic aid by international organizations and NGOs, as well as the provision of training and skill-building programs virtually by foreign and even domestic volunteer experts can be recommended based on the results of the current study.

According to the results, there is a bidirectional relationship between food insecurity and the QOL. Some recent food security literature have indicated the bidirectional relationship between food security and poor health outcome [22, 36–38, 59]. To our knowledge, this is the first study that found a bidirectional relationship between food security and QOL in the context of the refugee population, indicating its complex nature. Longitudinal studies are warranted to investigate such relationships in depth and over time, accounting for other potential factors. Also, the association of socioeconomic and sociodemographic factors with food insecurity after and before the Taliban takeover can be investigated by researchers in future studies.

All the suggestions presented above depend on the Taliban’s cooperation in communicating with international organizations and implementing policies to improve the food security of Afghan households. Without their collaboration, none of the recommendations of the present study, and even the suggestions of other studies could be implemented.

### Limitations and strengths

The data collection process was time-consuming, and the interviewers faced arduous obstacles during the process of gathering data, including long distances between the studied provinces, the lack of access to sufficient transportation facilities, and the concern of the families to respond to questions. Also, the highly dispersed households in the study areas and difficult access to them are other limitations of this study. Data on food security and quality of life before Taliban takeover depended on participants’ memory, which could introduce recall bias, but we used some strategies that might reduce recall bias include careful selection of the research questions, choosing an appropriate data collection method, and familiarizing questioners with how to ask questions. Finally, observational studies have a scientific weakness in that they can be used only to find associations between independent factors and responses, but alone they cannot establish causation. That does not diminish their importance. On the other hand, the present study is the first comparative research looking at the pre-Taliban and post-Taliban period that has evaluated quality of life and food security in these two periods. According to the study’s data, it is possible to provide practical policies to improve the quality of life and food security for the people of Afghanistan during the Taliban’s governance.

## Data Availability

The datasets used and/or analyzed during the current study available from the corresponding author on reasonable request.

## Nomenclature

FAO: Food and Agricultural Organization
FI: Food insecurity
HFIAS: Household Food Insecurity Access Scale
Max: Maximum
Min: Minimum
N: Number
NGOs: Non-Governmental Organizations
P: Probability
QOL: Quality of life
Std: Standard deviation
Sig: Significant level
SPSS V.25: Statistical Package for the Social Sciences, Version 25
TT: Taliban’s takeover
UN: United Nations
WFP: World Food Program
WHOQOL-100: WHO quality of life questionnaire with 100 questions
Z: Z-statistics

## Footnotes

1 In the WHOQOL-100 protocol, the following questions are used to calculate the overall quality of life: G1-How would you rate your quality of life? G2- How satisfied are you with the quality of your life? G3- In general, how satisfied are you with your life? G4- How satisfied are you with your health?
